# Using an oblique incident laser beam to measure the optical properties of stomach mucosa/submucosa tissue

**DOI:** 10.1186/1471-230X-9-64

**Published:** 2009-08-28

**Authors:** Hua Jiang Wei, Da Xing, Bo Hua He, Huai Min Gu, Guo Yong Wu, Xue Mei Chen

**Affiliations:** 1 MOE Key laboratory of Laser Life Science and Institute of Laser Life Science, South China Normal University, Guangzhou 510631, Guangdong Province, PR China; 2 Department of Surgery, the First Affiliated Hospital, Guangdong College of Pharmacy, Guangzhou 510224, Guangdong Province, PR China; 3 Department of Surgery, the First Affiliated Hospital, Sun Yat-Sen University, Guangzhou 510080, Guangdong Province, PR China; 4 Department of Ophthalmology, the First Affiliated Hospital, Sun Yat-Sen University, Guangzhou 510080, Guangdong Province, PR China

## Abstract

**Background:**

The purpose of the study is to determine the optical properties and their differences for normal human stomach mucosa/submucosa tissue in the cardiac orifice *in vitro *at 635, 730, 808, 890 and 980 nm wavelengths of laser.

**Methods:**

The measurements were performed using a CCD detector, and the optical properties were assessed from the measurements using the spatially resolved reflectance, and nonlinear fitting of diffusion equation.

**Results:**

The results of measurement showed that the absorption coefficients, the reduced scattering coefficients, the optical penetration depths, the diffusion coefficients, the diffuse reflectance and the shifts of diffuse reflectance of tissue samples at five different wavelengths vary with a change of wavelength. The maximum absorption coefficient for tissue samples is 0.265 mm^-1 ^at 980 nm, and the minimum absorption coefficient is 0.0332 mm^-1 ^at 730 nm, and the maximum difference in the absorption coefficients is 698% between 730 and 980 nm, and the minimum difference is 1.61% between 635 and 808 nm. The maximum reduced scattering coefficient for tissue samples is 1.19 mm^-1 ^at 635 nm, and the minimum reduced scattering coefficient is 0.521 mm^-1 ^at 980 nm, and the maximum difference in the reduced scattering coefficients is 128% between 635 and 980 nm, and the minimum difference is 1.15% between 890 and 980 nm. The maximum optical penetration depth for tissue samples is 3.57 mm at 808 nm, and the minimum optical penetration depth is 1.43 mm at 980 nm. The maximum diffusion constant for tissue samples is 0.608 mm at 890 nm, and the minimum diffusion constant is 0.278 mm at 635 nm. The maximum diffuse reflectance is 3.57 mm^-1 ^at 808 nm, and the minimum diffuse reflectance is 1.43 mm^-1 ^at 980 nm. The maximum shift Δx of diffuse reflectance is 1.11 mm^-1 ^at 890 nm, and the minimum shift Δx of diffuse reflectance is 0.507 mm^-1 ^at 635 nm.

**Conclusion:**

The absorption coefficients, the reduced scattering coefficients, the optical penetration depths, the diffusion coefficients, the diffuse reflectance and the shifts of diffuse reflectance of tissue samples at 635, 730, 808, 890 and 980 nm wavelengths vary with a change of wavelength. There were significant differences in the optical properties for tissue samples at five different wavelengths (*P *< 0.01).

## Background

Knowledge of optical properties for the human stomach mucosa/submucosa tissues in the visible and near infrared (NIR) wavelength range is of great importance in medical applications using light [1,2], for example, laser coagulation for treatment of early gastric cancer with intramucosal invasion, laser ablation therapy of the submucosal gastric cancer [3], photodynamic ablation therapy of early cancers of the stomach [4], gastrointestinal (GI) diagnosis by the standard white light endoscopy (WLE) and endoscopic diagnosis of premalignant gastrointestinal lesions by fluorescence endoscopic imaging and spectroscopy [5-7], and the recently developed optical coherence tomography (OCT) [8-10] has been reported to image the GI tissues in vitro and in vivo [11-13]. Because of more than 85% of all cancers originate in the epithelia lining the internal surfaces of the human body. The majority of such lesions are readily treatable if diagnosed at an early state [14]. Apart from conventional methods of cancer diagnosis [15-17], there is a need to develop new approaches that are simple, objective, and noninvasive.

The use of optical techniques for gastrointestinal diagnostic purposes relies on the capability to measure the optical properties of gastrointestinal tissue. In recent years, an increasing group of researchers has been interested in nonionizing, near-infrared (NIR) approaches for detecting and imaging diseased tissues. The proposed techniques range from continuous wave [18,19] to frequency-domain [20,21] or time-depended measurements of scattered light [22,23]. These techniques are based on the determination of optical properties of scattering media. The optical properties are represented by the absorption coefficient μ_a_, the scattering coefficient μ_s _and the anisotropy factor g. since the optical detecting and optical imaging are based on selective differences existing in optical properties of healthy and pathological tissues, it is particularly important to diagnostic purpose. For example, Laser-induced autofluorescence (LIAF) spectroscopy has been found to be a promising tool for early cancer diagnosis in gastrointestinal tract, including other organs [24,25]. Consequently tissue optical properties of healthy and pathological human gastrointestinal tissue are important for medical applications in diagnosis and therapy [26]. We focus in this paper on the optical properties of normal human stomach mucosa/submucosa tissue in the cardiac orifice at the visible and near-infrared wavelength range. The results were analyzed and compared from these experimental data we obtained.

### Theory

We utilize a simple two-source diffusion theory model of spatially resolved, steady-state diffuse reflectance [27]. When light enters a semi-infinite tissue, it will generally scatter a number of times before either being absorbed or escaping the tissue surface at a point other than its point of entry. The multiply scattered light that escapes is called diffuse reflectance. Wang and Jacques believe that for both normal and oblique incidence, the more accurate expression for the path length from the tissue surface to the positive point source is what it have been defined as 3D (D is the diffusion coefficient) rather than 1 mfp' (mfp' is the transport mean free path). These two cases were diagrammed in Ref.[28]. The diffuse reflectance profile for oblique incidence is centered about the position of the point sources, the shift Δx by finding the center of diffuse reflectance relative to the light entry point can be measured. As is the case for normal incidence, the diffusion theory model, when shifted by Δx, also agrees with Monte Carlo results outside of 1–2 mfp' from the center of diffuse reflectance, which, it is important to reiterate, is no longer at the point of entry as shown in Ref.[28]. The two-source model, with a depth of 3D instead of 1 mfp', gives the following expression [27,28]:(1)

which can be scaled arbitrarily to fit a relative reflectance profile that is not in absolute units. Where, μ_eff _is the effective attenuation coefficient, is defined as(2)

*ρ*_1 _and *ρ*_2 _are the distances from the two sources to the point of interest (the point of light collection; see Ref.[28]), and the boundary condition is included in the term A [28]:(3)

where(4)(5)

n_tissue _is the refractive index of the tissue, n_ambient _is the refractive index of the ambient, and n_rel _is the relative refractive index of the tissue-air interface. A laser beam is obliquely incident on the top face of the tissue sample, where, θ_tissue _is incident angle of the laser beam. D is the diffusion coefficient, it can be calculated from Δx(6)

where, Δx is the distance between the point of light incidence and the apparent center of diffuse reflectance. According to Lin et al [28] this diffusion constant is equal to(7)

with μ_s_' the reduced scattering coefficient, i.e. μ_s _(1-g), μ_a _the absorption coefficient. The optical properties, μ_a _and μ_s_' were solved from the expressions, and the expressions of μ_a _and μ_s_' are shown as follows(8)(9)

The method to determine tissue optical properties, μ_a _and μ_s_', need sample the relative diffuse reflectance profile at known positions from the light entry point, and need calculate Δx and D, and need perform a nonlinear least-squares fit with the Levenberg-Marquardt method [29-31] on (1) to determine μ_eff_, and then need solve for μ_a _and μ_s_' from the expressions. The method was detailedly shown in Ref.[28].

## Methods

### Sample preparation

Normal human stomach mucosa/submucosa tissues in the cardiac orifice were investigated in this study. Tissue samples were taken from 12 normal human stomachs in the cardiac orifice were determined from histological examination, immediately after excision the tissues. Each removed stomach sample was immediately rinsed briefly in saline to remove surface excess blood and peeled off surface fats, was placed in a bottle with saline as soon as possible, and was stored in a refrigerator at -70°C. From tissue samples a total of 12 normal stomach mucosa/submucosa tissue samples, with a mean thickness of (10.32 ± 0.26) mm, were used within at most 24 h after remove. The thickness of each sample was measured and recorded with a vernier caliper with 0.02 mm error. All tissue samples were respectively taken out from the refrigerator before measurement, were placed on experimental desk at the room temperature of 20°C for one hour, and then all thawing tissue samples were measured in turn using an oblique incident laser beam and CCD camera, respectively.

### Diffuse reflectance measurements of tissue

**Figure 1:**
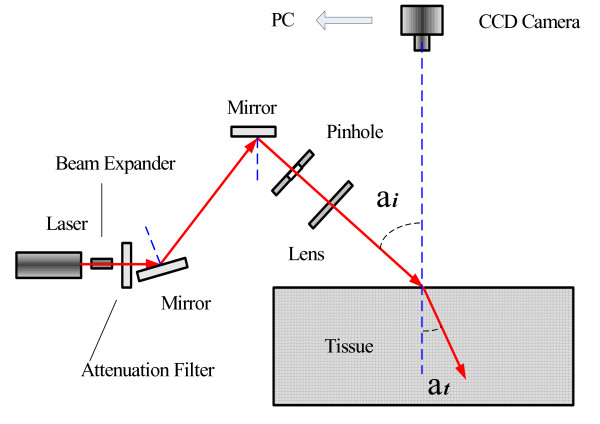
**Schematic diagram of experimental set-up as used for the measurement of the diffusion constant and the distribution of diffuse reflection light**.

Figure 1 shows a schematic diagram of the experimental setup that is used to measure the relative profile of diffuse reflectance, and table 1 shows information about light source on the experiment. The tissue samples were illuminated with collimated light from 635, 730, 808, 890 and 980 nm wavelength of laser, respectively. The output of all laser light were expanded by the beam expander of 25 times, and then were attenuated (to a power at most 5 mW) by the light attenuators, and were reflected by the mirrors, were passed through a 2 mm pinhole and a 35.2 mm focus of lens, and then the obliquely incident on the top face of the stomach mucosa/submucosa tissue sample at a 45 degree angle between laser axis and the normal to the tissue surface (α_i _= 45°), respectively. A small piece of transparent ruler (with millimeter gradations) was placed onto the sample surface for scale, and a certain graduation of the ruler was leveled to the center portion of the point of incidence of the laser beam, and the graduation is designated as the origin of the x-coordinate. From the top of the sample a reflectance pattern can be observed. This pattern is imaged on a 795×596 pixel two-dimensional Charge Coupled Device (CCD) detector (Nikon, Cool Pix, 995, Japan). The incident beam can be observed as the most intense area in the image. Because, the laser beam was oblique to the surface the reflectance pattern was asymmetrical near the point of incidence, but the diffuse reflectance far from the source formed concentric circles, approximately, and the distance between the origin of the x-coordinate and the centre of the concentric circles is the distance Δx, and the centre of the concentric circles is also calculated. From the distance Δx the diffusion constant can be calculated using (6), with D the diffusion constant in mm, Δx the distance in mm. This test consisted of repeating ten times reflectance measurements, and the measured results were reproducible for a specific sample at specific wavelength. For each test, the positions of the spot of incident light on the sample surface were altered to decrease the effect of the tissue heterogeneity on the reflectance measurements, and each test at each laser wavelength was performed in the same condition of experimentation, and the exposure time was set at 800 ms. A total of eleven tissue samples were used for the measurements in vitro. The CCD data acquisition were controlled by a computer for the purpose. Data processing and analysis of the data files were performed using custom software written in Matlab (Matlab, Mathworks Incorporated, Massachusetts).

**Table 1:** Kinds, model of laser and power output of using light source on the experiment

Light source	Model	Power output
635 nm wavelength of diode laser	nLIGHT, USA, model NL-FBA-2.0–635	P ≤ 5 mW
730 and 890 nm wavelengths of Ti:S ring laser	COHERENT, USA, model 899-05	P ≤ 5 mW
808 nm wavelength of diode laser	nLIGHT, USA, model NL-FCA-20–808	P ≤ 5 mW
980 nm wavelength of diode laser	nLIGHT, USA, model NL-FCA-30–980	P ≤ 5 mW

### Statistical analysis

Optical parameters of biological tissue samples were expressed as the mean ± SD, were demonstrated by a Student *t*-test, and were considered significant at *p *values < 0.01. The SPSS10 was used for the statistical analysis.

## Results

**Figure 2:**
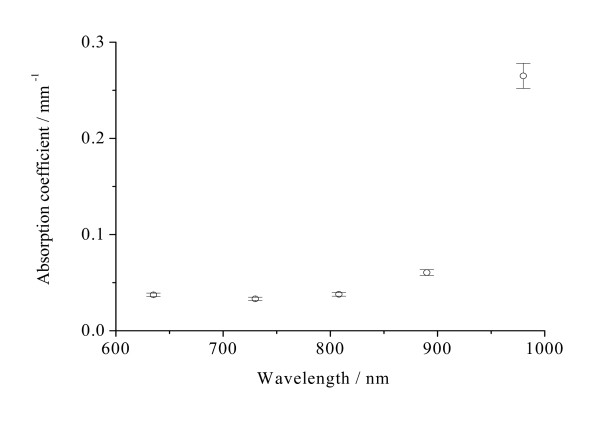
**The wavelength dependence of the absorption coefficients μ_a _of normal stomach mucosa/submucosa tissues in the cardiac orifice**. The blank dots correspond to the averaged absorption coefficients and the vertical lines show the SD values.

**Figure 3:**
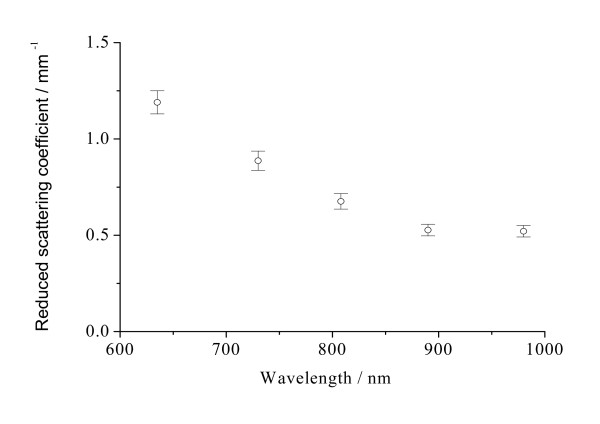
**The wavelength dependence of the reduced scattering coefficients μ_s_' of normal stomach mucosa/submucosa tissues in the cardiac orifice**. The blank dots correspond to the averaged reduced scattering coefficients and the vertical lines show the SD values.

**Figure 4:**
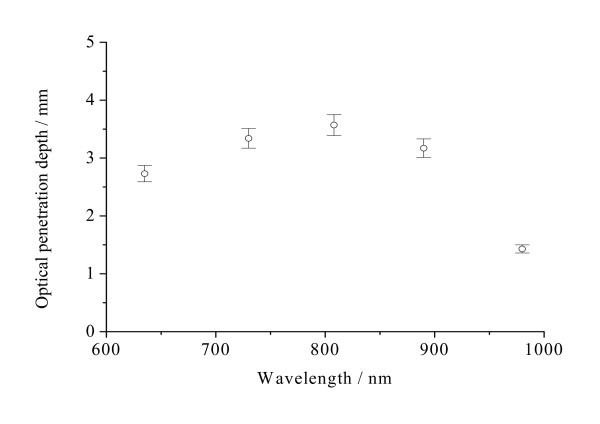
**The optical penetration depths δ of normal stomach mucosa/submucosa tissues in the cardiac orifice at 635, 730, 808, 890 and 980 nm**. The blank dots correspond to the average optical penetration depths and the vertical lines show the SD values.

**Figure 5:**
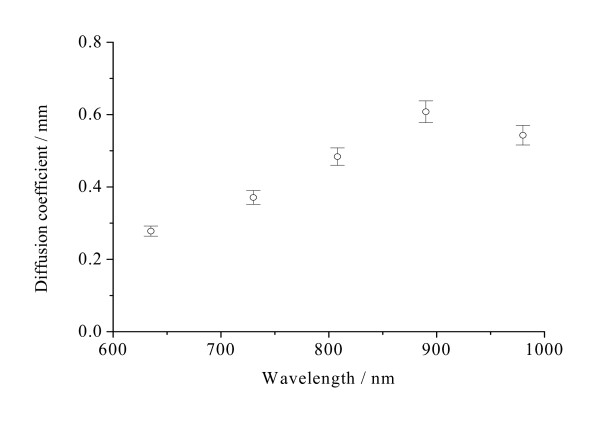
**The diffusion coefficients *D *of light into normal stomach mucosa/submucosa tissues in the cardiac orifice at 635, 730, 808, 890 and 980 nm**. The blank dots correspond to the average diffusion coefficients and the vertical lines show the SD values.

**Figure 6:**
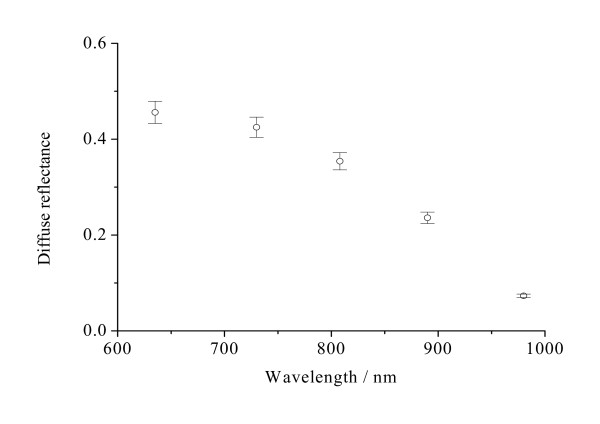
**The diffuse reflectance R_∞ _of normal stomach mucosa/submucosa tissues in the cardiac orifice at 635, 730, 808, 890 and 980 nm**. The blank dots correspond to the average diffuse reflectance and the vertical lines show the SD values.

**Figure 7:**
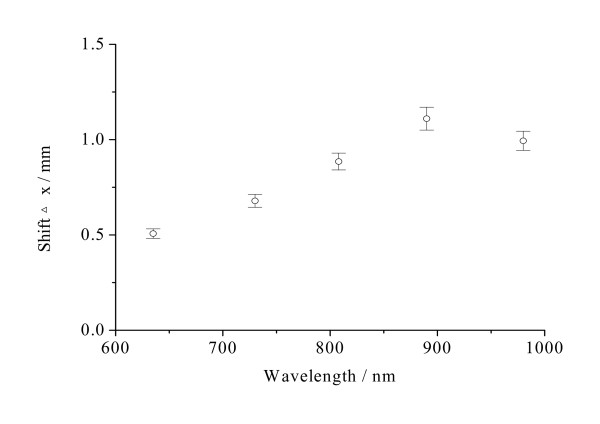
**The shift Δx of diffuse reflectance of normal stomach mucosa/submucosa tissues in the cardiac orifice at 635, 730, 808, 890 and 980 nm**. The blank dots correspond to the average shift Δx of diffuse reflectance and the vertical lines show the SD values.

The optical properties are expressed as the mean ± SD for all measurements for the samples. Figures 2, 3, 4, 5, 6 and 7 present the wavelength dependence of the absorption coefficients, the reduced scattering coefficients, the optical penetration depths, the diffusion coefficients, the diffuse reflectance and the shifts of diffuse reflectance for normal stomach mucosa/submucosa tissues in the cardiac orifice at five different wavelengths of laser, respectively. The vertical lines correspond to the values of standard deviation (SD), which is determined by a Student *t*-test, and error bars appear at 635, 730, 808, 890 and 980 nm wavelengths of laser for clarity and represent one standard deviation in the μ_a_, μ_s_', δ, D, R_∞ _and Δx values.

## Discussion

The optical properties of a biological tissue depend on its biochemical composition and its cellular and subcellular structure. In the visible and near-infrared range, the absorption properties are related to the concentration of chromophores, such as oxyhemoglobin and deoxyhemoglobin, fat and water [32]. Such chromophores vary significantly with tissue metabolism [33]. The scattering properties are related to the size distribution of cells and organelles, which are parameters used to differentiate normal from abnormal tissues in standard histopathology [34]. Therefore optical measurements have a strong potential for the development of noninvasive *in vivo *medical diagnostic tools, often called "optical biopsy". Such techniques should significantly improve the efficiency of biopsies or help in determining the tumor margins in a surgical field. According to our experimental data, the absorption coefficients, the reduced scattering coefficients, the optical penetration depths, the diffusion coefficients, the diffuse reflectance and the shifts of diffuse reflectance for normal stomach mucosa/submucosa tissues in the cardiac orifice at 635, 730, 808, 890 and 980 nm were determined in vitro. In our study, it is interesting to note the optical properties measured and their differences for the tissue samples at five different laser wavelengths. We believe the optical properties should help to pathological diagnosis and medical treatment for malignant or premalignant gastrointestinal mucosa with ease by using optical methods.

Figure 2 and Figure 3 show the absorption coefficients and the reduced scattering coefficients of tissue samples at five different laser wavelengths, respectively. From Figure 2 and Figure 3, it can be seen that the absorption coefficients for tissue samples increase with the increase of laser wavelengths, except for the absorption coefficient at 730 nm, and the reduced scattering coefficients for tissue samples decrease with the increase of laser wavelengths. There were significant differences in the absorption coefficients at five different laser wavelengths (*P *< 0.01). The maximum and the minimum absorption coefficients are 0.265 mm^-1 ^at 980 nm and 0.0332 mm^-1 ^at 730 nm, respectively. The maximum and the minimum differences of the absorption coefficients are 698% between 730 and 980 nm and 1.61% between 635 and 808 nm, respectively. There also were significant differences in the reduced scattering coefficients at five different laser wavelengths (*P *< 0.01). The maximum and the minimum reduced scattering coefficients are 1.19 mm^-1 ^at 635 nm and 0.521 mm^-1 ^at 980 nm, respectively. The maximum and the minimum differences of the reduced scattering coefficients are 128% between 635 and 980 nm and 1.15% between 890 and 980 nm, respectively.

Figure 4 shows that the optical penetration depths for tissue samples vary with the increase of laser wavelengths. There were significant differences in the optical penetration depths at five different laser wavelengths (*P *< 0.01). The maximum and the minimum optical penetration depths are 3.57 mm at 808 nm and 1.43 mm at 980 nm, respectively. The maximum and the minimum differences of the optical penetration depths are 150% between 808 and 980 nm and 5.36% between 730 and 890 nm, respectively. From Figure 5, it can be seen that the diffusion coefficients for tissue samples vary with the increase of laser wavelengths. There also were significant differences in the diffusion coefficients at five different laser wavelengths (*P *< 0.01). The maximum and the minimum diffusion coefficients are 0.608 mm^-1 ^at 890 nm and 0.278 mm^-1 ^at 635 nm, respectively. The maximum and the minimum differences of the diffusion coefficients are 119% between 635 and 890 nm and 12.0% between 890 and 980 nm, respectively. Figure 6 shows that the diffuse reflectance for tissue samples decrease with the increase of laser wavelengths. There were significant differences in the diffuse reflectance at five different laser wavelengths (*P *< 0.01). The maximum and the minimum diffuse reflectance are 0.456 at 635 nm and 0.0732 at 980 nm, respectively. The maximum and the minimum differences of the diffuse reflectance are 523% between 635 and 980 nm and 7.29% between 635 and 730 nm, respectively. From Figure 7, it can be seen that the shift Δx of diffuse reflectance for tissue samples vary with the increase of laser wavelengths. There also were significant differences in the shift Δx of diffuse reflectance at five different laser wavelengths (*P *< 0.01). The maximum and the minimum shift Δx of diffuse reflectance are 1.11 mm at 890 nm and 0.507 mm at 635 nm, respectively. The maximum and the minimum differences of the shift Δx of diffuse reflectance are 119% between 635 and 890 nm and 11.7% between 890 and 980 nm, respectively.

There are significant differences in the optical properties of the tissue samples between different wavelengths of laser (*P *< 0.01). Bashkatov, et al.[35] and Holmer et al.[36] have reported the optical properties of gastric tissue by different optical measurement methods, our data that the wavelength dependence of the absorption coefficient, the reduced scattering coefficient and the optical penetration depth of human stomach wall mucosa are very similar to compare the data of Bashkatov, et al. and Holmer et al. with our data in the spectral range from 600 to 1000 nm.

## Conclusion

In conclusion, the results reported here indicate that differences in the optical properties, namely, the absorption coefficients, the reduced scattering coefficients, the optical penetration depths, the diffusion coefficients, the diffuse reflectance and the shifts of diffuse reflectance for normal stomach mucosa/submucosa tissues in the cardiac orifice at 635, 730, 808, 890 and 980 nm are significant in vitro (*P *< 0.01), and the potential and promise of using an oblique incident laser beam to measure the optical properties of tissue for clinical studies. Tissues of various pathologies have differing optical tissue properties, and tissues of different places for normal human stomachs have differing optical tissue properties [2]. The preliminary results presented can be used for the development of optical technologies and can be useful in earlier diagnosis, photodynamic and photothermal therapy in the gastrointestinal tract.

## Abbreviations

NIR: near infrared; GI: gastrointestinal; WLE: white light endoscopy; OCT: optical coherence tomography; LIAF: laser-induced autofluorescence; mfp': the transport mean free path; D: the diffusion coefficient; SD: standard deviation

## Competing interests

The authors declare that they have no competing interests.

## Authors' contributions

HW has been involved in the design and conception of the study, supervision of the work, acquisition and analysis of data, and writing the manuscript. DX contributed in the design of the study, supervised the study. BH participated in the histological studies, carried out gastric biopsies, and performed the statistical analysis. HG coordinated the study. GW participated in the histological studies, and carried out gastric biopsies, and performed the statistical analysis. XC participated in the histological studies, and carried out gastric biopsies, and performed the statistical analysis. All authors read and approved the final manuscript.

## Pre-publication history

The pre-publication history for this paper can be accessed here:

http://www.biomedcentral.com/1471-230X/9/64/prepub
